# The Effectiveness of a New Hemostatic Agent (Ankaferd Blood Stopper) for the Control of Bleeding following Tooth Extraction in Hemophilia: A Controlled Clinical Trial

**DOI:** 10.4274/tjh.2012.0036

**Published:** 2013-03-05

**Authors:** Hakkı Oğuz Kazancıoğlu, Onur Çakır, Gülsüm Ak, Bülent Zülfikar

**Affiliations:** 1 Bezmialem Vakıf University, School of Dentistry, Department of Oral and Maxillofacial Surgery, İstanbul, Turkey; 2 İstanbul University, School of Dentistry, Department of Oral and Maxillofacial Surgery, İstanbul, Turkey; 3 İstanbul University, Cerrahpaşa School of Medicine, Department of Pediatric Hematology and Oncology, İstanbul, Turkey

**Keywords:** Ankaferd Blood Stopper, Oral surgery, Hemophilia, Hemostasis

## Abstract

**Objective:** To assess the hemostatic efficacy of a new local hemostatic agent, Ankaferd Blood Stopper (ABS), for the control of bleeding following tooth extraction in hemophiliacs.

**Materials and Methods:** Simple tooth extractions were performed in 27 hemophilia A patients. In the treatment group (n=17) local hemostasis was achieved via application of ABS to the extraction sockets, whereas in the control group (n=10) local hemostasis was achieved via direct packing with gauze.

**Results:** In all, 57 (21 primary and 36 permanent) teeth extractions were performed in 27 hemophilia A patients. There were no significant differences in age or factor VIII level distribution between the 2 groups (p>0.05). The most significant clinical difference between the groups was associated with the use of ABS; those in the treatment group had significantly shorter duration of bleeding (p=0.002).

**Conclusion:** This is the first study to evaluate the efficacy of ABS for the control of bleeding following tooth extraction in hemophiliacs. ABS can be considered an alternative local hemostatic agent for reducing clotting factor concentrates in hemophilia patients.

**Conflict of interest:**None declared.

## Abstract (tr)

**Amaç:** Yeni bir hemostatik ajan olan Ankaferd Blood Stopper (ABS)’ın hemofili hastalarında diş çekimi sonrası kanama kontrolüne etkisini değerlendirmek.

**Gereç ve Yöntemler:** 27 hemofili hastasının basit diş çekimleri gerçekleştirildi ve 17 hastaya ABS uygulandı. Kontrol grubunda (n=10) lokal hemostaz gaz tampon uygulanarak sağlandı. ABS ile tedavi edilen grupta (tedavi grubu) lokal hemostaz ABS’nin çekim soketine uygulanması ile sağlanmıştır.

**Bulgular:** 27 hemofili hastasından toplam 57 (21 süt ve 36 sürekli) diş çekimi gerçekleştirildi. Gruplar arasında yaş ve Faktör VIII seviyeleri açısından istatiksel olarak anlamlı fark bulunamamıştır (p>0,05). Gruplar arasında en önemli klinik farklılık ABS kullanımı ile ilişkilidir. ABS uygulanan grubun kanama süresi daha düşüktür (p=0,002).

**Sonuç:** Bu çalışma hemofili hastalarında diş çekimi sonrasında ABS’nin etkinliğini değerlendiren ilk çalışmadır. ABS hemofili hastalarında pıhtılaşma faktörü uygulama oranını azaltmak için alternatif bir hemostatik ajan olarak düşünülebilir.

## INTRODUCTION

Hemophilia is an X-linked hereditary disorder with male predominance and a frequency of approximately 1/10,000 births. Hemophilia A occurs due to a deficiency of factor (F) VIII and hemophilia B occurs due to a deficiency of FIX [1,2]. According to factor activity, hemophilia can be classified as severe (<1% of normal), moderate (1%-5% of normal), or mild (5%-40% of normal) [1,3]. 

The deficient factors should be complemented with factor concentrate when hemophilia patients undergo surgical procedures. In cases of minor surgical intervention (tooth extraction, supragingival periodontal treatment) and major surgical intervention (surgical tooth extraction with complication, subgingival periodontal treatment) the desired foctor levels should be approximately >50 IU/dL and >80 IU/dL, respectively [4].

Due to the lack of hemostasis protocols, interventions to prevent bleeding are delayed, and dentists usually avoid treating hemophilia patients because of the high risk of bleeding and inadequate factor support. As a result of these conditions and inadequate preventive dentistry, tooth extractions and odontogenic cyst development are inevitable in hemophiliacs. Conventional hemostatic agents are expected to aid a patient’s coagulation system in rapidly developing an occlusive clot via platelet adhesion, platelet activation, and blood coagulation. Various local hemostatic agents have been used in oral and maxillofacial surgical practice. Hemostatic agents vary in effectiveness, cost, and ease of use. The ideal oral surgery hemostatic agent should be safe, well-tolerated, bacteriostatic, preformed for operator convenience, packaged, sterile, single-use, and able to remain where applied, dissolve during the first post-surgery week, and integrate with current oral surgery treatment protocols without the need for special procedures. What is needed is an absorbable hemostatic agent that can be successfully used in the treatment of major solid organ injuries and does not depend on platelet and coagulation factors for its hemostatic efficacy. To date, no hemostatic device has completely met these criteria [3,4,5].

Ankaferd Blood Stopper (ABS) (Ankaferd Health Products, Ltd., İstanbul, Turkey) is a new hemostatic agent comprising the following plant extracts: Urtica dioica (0.06 mg/mL), Vitis vinifera (0.08 mg/mL), Glycyrrhiza glabra (0.07 mg/mL), Alpinia officinarum (0.07 mg/mL), and Thymus vulgaris (0.05 mg/mL). Each of these plant extracts has some effects on the endothelium, blood cells, angiogenesis, cellular proliferation, vascular dynamics, and cell mediators. Although the basic mechanism of action of ABS remains unclear, it appears to cause the formation of an encapsulated protein network representing focal points for vital erythrocyte aggregation. The ABS-induced protein network that forms with blood cells, particularly erythrocytes, covers the primary and secondary hemostatic system without disturbing individual coagulation factors. ABS also upregulates the GATA/FOG transcription system, affecting erythroid functions and urotensin II. These data have been obtained via MALDI-TOF proteomic molecular analysis, cytometric array, transcription analysis, and scanning electron microscopy examination in in vitro and in vivo research settings [6,7,8].

Exaggerated bleeding, primarily in patients with hereditary or acquired hemorrhagic diathesis, is a challenging issue in daily dental practice; therefore, ABS may be useful for treating patients with bleeding disorders. Since the Turkish Ministry of Health authorized the use of ABS for the management of dental bleeding, ABS has been added to protocols for the prevention and treatment of prolonged hemorrhaging due to dental procedures. The present study aimed to evaluate the effects of ABS on the duration of bleeding following dental extractions in hemophiliacs. To the best of our knowledge this is the first study on the use of ABS on dental extraction sockets in hemophilia patients.

## MATERIALS AND METHODS

**Patients and Design**

This cross-sectional controlled clinical trial included 27 male hemophilia A patients that required dental extractions. The mean FVIII level in the patients was 2.41±2.58% (range: 0%-9%). The participants were assigned to the control group (n=10) or treatment group (n=17) regardless of disease severity. Each patient underwent an initial consultation to establish a dental treatment plan. All mild to moderate hemophilia A patients with severe dental caries, dental abscess, and prolonged retention of deciduous teeth that required tooth extraction were candidates for inclusion in the study. Patients that had any psychological disease or systemic disease, such as diabetes mellitus and hypertension, were excluded from the study. 

In total, 32 patients met the study’s inclusion criteria; however, 3 did not agree to participate and were thereby excluded. The study began with 29 patients, but 2 were subsequently excluded because they did not follow the suggested protocol. Before dental extraction all patients received standard factor replacement therapy according to our hospital’s guidelines (Table 1). Only those in the treatment group received ABS, which was applied locally after dental extraction. Demographic data, factor levels, duration of bleeding, dental treatments, and pre- and postoperative additional factor infusion were recorded (Table 2). The study was conducted in accordance with the Declaration of Helsinki. Before commencement of the study, written informed consent was obtained from each patient or their parents. Each of the patients’ physicians was consulted. 

All of the hemophilia patients underwent our clinic’s dental treatment protocol (Table 1). Factor replacement therapy was performed 1 h prior to tooth extraction, according to patient weight. Recombinant human factor VIIa (rFVIIa; NovoSeven^®^; Novo Nordisk A/S, Bagsværd, Denmark) (90 µg/kg) was administered 1 h prior to tooth extraction for bleeding control in patients who had inhibitor against factor VIII, and this procedure was applied again at hours 3 and 6 after surgery. In addition, these patients used tranexamic acid (30/mg/kg/day) orally for 5-7 days. All extractions were performed under local anesthesia using a 2% ultracaine-DS ampule consisting of 40 mg articaine HCl and 0.006 mg/mL epinephrine HCl (Sanofi/Aventis, Germany). Patients were sent to their hematologists for postoperative factor replacement therapy and bleeding control. 

All tooth extractions were performed by the same qualified oral surgeon using forceps and/or elevators. The extractions were carried out in the least traumatic way possible and the extraction sites were carefully removed. In the treatment group, local hemostasis was achieved via local application of ABS on the extraction sockets. ABS was topically applied via high-pressure homogeneous spraying into the cavity. If oozing persisted, extra doses of ABS were topically applied. In the control group, local hemostasis was achieved via direct packing with gauze.

**Bleeding assessment**

An investigator blinded to the treatment measured the duration of bleeding in seconds, from the time bleeding started to cessation of free-flowing blood from outside the cavity, using a digital chronometer.

**Postoperative care**

The patients were given postoperative instructions. On the day of surgery the patients were followed for hemorrhage during the first 2 h after extraction. Follow-up appointments were then scheduled for 1, 3, and 7 days after surgery. In cases of persistent bleeding, hematologists were consulted for treatment. Single and multiple (up to 4 teeth) extractions were considered low-risk interventions, and these patients were classified into a low-risk subgroup. In total, 57 (21 primary and 36 permanent) teeth extractions were performed in 27 hemophilia patients. No bleeding complications occurred during the extractions. All the surgical procedures were simple extractions that did not require elevation of a mucoperiosteal flap.

**Statistical analysis**

Statistical analysis (descriptive and analytical) of the data was performed using MS Office Excel and SPSS v.15.0 for Windows. The Mann-Whitney U test was used to evaluate the differences between the groups. Data are presented as percentage, mean ± SD, and median, IQR (interquartile range), where appropriate. The level of statistical significance was set at p<0.05.

## RESULTS

The study included 27 hemophilia A patients. The treatment group consisted of 17 male patients with a mean FVIII level of 2.5% (range: 0%-8.7%) and mean age of 20.35±11.41 years (range: 5-46 years). The control group included 10 male patients with a mean FVIII level of 2.2% (range: 0%-9%) and mean age of 21.10±13.43 years (range: 8-42 years). There were no significant differences in age, FVIII level distribution, or FVIII consumption before and after surgery between the 2 groups (p>0.05). The most significant clinical difference between the groups was associated with the use of ABS; those in the treatment group had significantly shorter duration of bleeding (Table 3). 

Local hemostasis and late bleeding were the criteria used to compare the treatment and control groups. Complete hemostasis was immediately obtained in both groups, except for 1 patient in the treatment group who had inhibitors (26 BU) against FVIII. This patient was treated with additional recombinant factor VIIa infusion. Patients in the treatment group were administered 1-2 mL of ABS; in most cases 1 mL of ABS was sufficient to control bleeding.Median duration of bleeding in the treatment group was 70 s (IQR: 55–79.5 s) versus 101 s (IQR: 77–122.5 s) in the control group and the difference was significant (p=0.002) (Table 3). Late bleeding was not observed after surgery. At the last follow-up, none of the patients had extraction socket wound infection. Delayed wound healing was observed only in an 8-year-old patient in the control group. No allergic reactions or side effects were attributed to the use of ABS.

## DISCUSSION

Hemophilia A is a secondary hemostasis disorder. To prevent spontaneous, traumatic, and surgical bleeding hemophilia patients require replacement of the missing clotting factor. This is especially true for hemophilia patients living in newly industrialized countries who have bad oral hygiene due to the fear of bleeding, nutritional deficiencies, and inadequate factor support. Due to the fact that hemostasis protocols cannot be used to determine a patient’s coagulation factor level, interventions to prevent bleeding are delayed. As a result of these conditions, it is necessary to employ a tooth extraction protocol for such patients. In patients undergoing oral surgery guidelines for the management of coagulation disorders recommend not disrupting the anticoagulation regimen, providing that the level of anticoagulation is within therapeutic levels according to the international normalized ratio [9,10,11].

The literature describes various dental treatment protocols that result in a remarkable reduction in the number of bleeding episodes following oral procedures, including the use of oral antifibrinolytic agents, systemic hemostatic replacement therapy, and use of local hemostatic agents [12,13]. The primary benefits of local hemostatic agents are a life-saving reduction in hemorrhaging caused by trauma, a reduction in factor dependency, a reduction in the cost of treatment, and rapid control of hemorrhaging, which reduces patient anxiety related to uncontrollable bleeding [14]. A retrospective study that included 63 patients with von Willebrand disease reported that there was no difference in the frequency of bleeding complications following dental extraction between patients that received only local therapy with tranexamic acid and fibrin glue, and those that received either desmopressin or FVIII/von Willebrand factor concentrates [15]. Hewson et al. [11] performed 113 dental extractions in 50 patients without previous infusion of clotting factor concentrates, and they reported that there were no severe bleeding complications during a follow-up period of 8 days and that only 4 patients required repeated concentrate infusion to control bleeding. They emphasized the role of local hemostatic techniques, such as careful incision procedures, use of tranexamic acid to fill the extraction sockets and as mouthwash, and use of absorbable hemostats and resorbable sutures. Mancuso [13] also reported that minimal infusion of factor replacement in dental treatment procedures could be performed via application of local hemostatic agents.

ABS is a folkloric medicinal plant extract that has been approved for the management of dental surgery and external hemorrhaging [8,16], and it is proven to be safe, efficacious, sterile, and nontoxic [17,18,19,20,21]. Goker et al. [7] reported that the ABS-induced network formation is correlated with blood protein and red blood cell functions. They also reported that ABS’s basic mechanism of action appears to be the formation of an encapsulated protein network that provides focal points for erythrocyte aggregation. Blood cells also aggregated and participated in the network formation, with the erythrocytes forming a mass. They also observed that plasma fibrinogen, total protein, albumin, and globulin levels decreased in response to ABS. This mechanism of ABS represents an advantage over other local hemostatic agents. Cipil et al. [17] reported that ABS had in vivo hemostatic actions that may be therapeutic for the clinical management of patients with deficient primary hemostasis. Furthermore, they reported that ABS exhibited a hemostatic effect via modulation of platelet functions. Oner et al. [16] applied FVIII inhibitor bypass activity, cyclophosphamide, prednisolone, and then ABS to treat persistent bleeding due to circumcision in a pediatric hemophiliac with inhibitor; only ABS effectively controlled the bleeding. 

Baykul et al. [22] investigated the effect of topical application of ABS on hemorrhagic diathesis in 4 patients following different dental procedures. In most of the patients ABS effectively controlled bleeding within 10-20 min following dental surgery. In the present study, ABS was observed to be an effective and safe alternative hemostatic agent. In the treatment group only 1 patient (a 32-year-old severe hemophilia A patient (0%) who had inhibitors against factor VIII (26 BU)) had prolonged postsurgical bleeding, which was stopped with reapplication of ABS and rFVIIa (hours 3 and 6). This patient had 3 teeth extracted at the same time, and ABS was applied with rFVIIa before and after extraction. We think that the bad fibrin formation was caused by the patient’s poor oral hygiene and multiple tooth extractions. No bleeding complication was observed in another patient with inhibitor (an 11-year-old severe hemophilia A patient (0.9%) (1.1 BU)). Inadequate wound healing occurred in an 8-year-old patient in the control group that had severe hemophilia A (0%). We think that this patient’s postoperative bleeding was caused by inadequate postoperative care, because the patient did not fully comply with the study’s protocol.

In the present study, ABS significantly reduced the duration of post-oral surgery bleeding. Based on the present findings, we think that ABS is effective, safe, and easy to use. Additionally, the blood coagulation process is driven by protein agglutination and ABS stimulates formation of an encapsulated protein network that provides space for erythrocyte aggregation in the injured vascular area. Furthermore, ABS also interacts with fibrinogen and other blood proteins. ABS has no effect on coagulation factor levels; therefore, ABS can be used in patients with deficient primary hemostasis and/or secondary hemostasis, including patients with disseminated intravascular coagulation [23]. In terms of cost-effectiveness, clotting factor concentrates are costly components of hemostatic therapy in patients with bleeding disorders; a reduction in their use will directly reduce the cost of therapy. Financial concerns have forced researchers to study ancillary therapies, such as new techniques or hemostasis protocols that might provide an opportunity to identify something that would be closer to optimal replacement therapy by using lower doses. In Turkey the cost of 1000 IU of FVIII and 1 mg of rFVIIa is approximately $555 and $722, respectively, whereas 1 mL of ABS cost only approximately $6.

In conclusion, to the best of our knowledge the present study is the first to evaluate the efficacy of ABS for the control of bleeding following tooth extraction in hemophilia patients. ABS was observed to effectively achieve hemostasis in the treatment group. Additional controlled trials are needed to further evaluate the effectiveness of ABS as an alternative local hemostatic agent in hemophilia patients, without the use clotting factor concentrates. 

**Conflict of Interest Statement**

The authors of this paper have no conflicts of interest, including specific financial interests, relationships, and/ or affiliations relevant to the subject matter or materials included.

## Figures and Tables

**Table 1:**
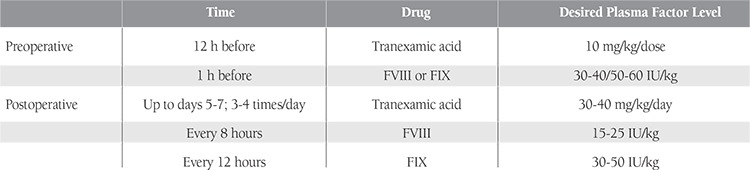
Hemostasis protocol.

**Table 2:**
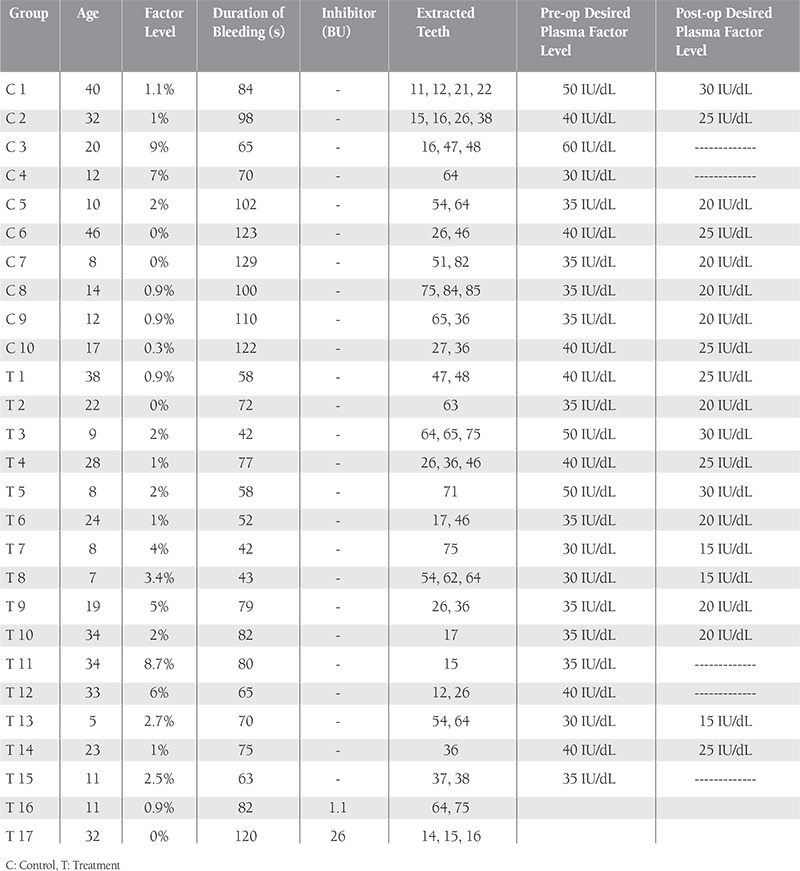
Demographic, medical, and dental extraction characteristics of the hemophilia A patients.

**Table 3:**
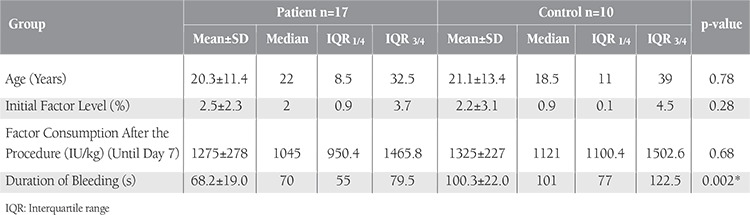
Age, initial Factor VIII level, Factor VIII consumption after the procedure, and the duration of bleeding variables for the patient group and the control group.
